# Supplementary material for: Immaturin-Nuclease as a Model System for a Gene-Programmed Sexual Development and Rejuvenescence in Paramecium Life History
Source: Microorganisms. 2022 Dec 28;11(1):82. doi: 10.3390/microorganisms11010082 (PMC9865852; doi:10.3390/microorganisms11010082)

Figure S1. Multiple amino acid sequence alignment of Immaturin.

Amino acid sequence alignments of immaturin and its orthologs were generated using MUSCLE with the MEGA5 program. Outlined and shaded text represent at least 80% identical (dark shading) or similar (light shading) chemical types of amino acid residues. Accession numbers for species names or IDs used in ParameciumDB are indicated. Paramecium IDs begin with PCAUDP, GSPATP, and PMMNP for *P. caudatum*, *P. tetraurelia*, and *P. multimicronucleatum* proteins, respectively.

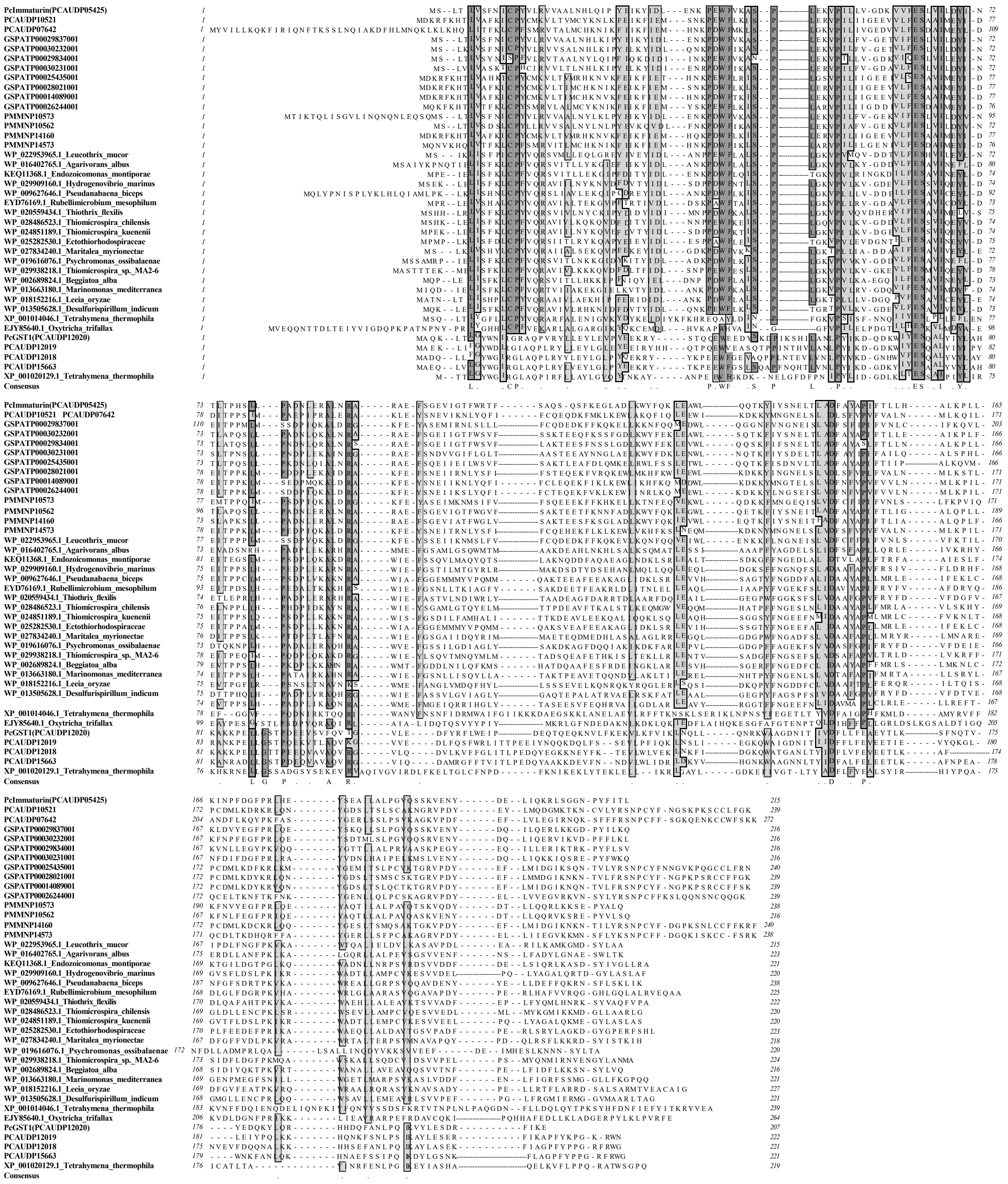

Supplement: Supplementary file 1 [file microorganisms-11-00082-s001.zip › microorganisms-2117495-supplementary.pdf]
